# Linkages between Soil Security and One Health: implications for the 2030 Sustainable Development Goals

**DOI:** 10.3389/fpubh.2024.1447663

**Published:** 2024-09-18

**Authors:** Tom Swan, Alex McBratney, Damien Field

**Affiliations:** ^1^The School of Life and Environmental Sciences, The University of Sydney, Camperdown, NSW, Australia; ^2^Sydney Institute of Agriculture, The University of Sydney, Camperdown, NSW, Australia

**Keywords:** Soil Security, One Health, planetary health, Soil Health, research linkages, sustainable development goals, global existential challenges

## Abstract

Soil provides multiple and diverse functions (e.g., the provision of food and the regulation of carbon), which underpin the health of animals, humans, the environment and the planet. However, the world’s soils face existential challenges. To this end, the concept of Soil Security was developed, compelled to: “maintain and improve soils worldwide so that they can continue to provide food, fiber and fresh water, contribute to energy and climate sustainability and help to maintain biodiversity and the overall protection of ecosystem goods and services.” In parallel, the concept of One Health likewise works across the human–animal–environment interface, highly relevant for the goals of Soil Security. In this review, we evaluated the roles which both the Soil Security and One Health concepts have served in the literature between 2012 and 2023 and explore the potential linkages between both concepts. We outline that both concepts are used in disparate fields, despite considerable overlap in aims and objectives. We highlight the Soil Health concept as a potential connector between Soil Security and One Health. Overall, we argue that both Soil Security and One Health are highly complementary fields of scientific inquiry with solid leverage for translation into policy and practice. However, there is a need to define One Health dimensions, as has been done for Soil Security. As such, we proffer five measurable dimensions for One Health, the “5Cs”–Capacity, Condition, Capital, Connectivity and Codification–to allow for an overall measure of One Health. Finally, we advocate for a biosphere-focused framework to collectively make progress toward the 2030 Sustainable Development Goals and other global existential challenges.

## Introduction

At the core of the 2030 United Nations (UN) Sustainable Development Goals (SDGs) agenda for sustainable development are 17 goals which, if implemented, should provide a relatively egalitarian and salubrious world to live in. Soils play an important role, directly and indirectly, to at least 12 of the 17 SDGs, most notably to SDG2 (Zero Hunger) by supporting food security and nutrition, in addition to supporting Climate Action (SDG13) and Sustaining Life on Land (SDG15) [as discussed comprehensively in (1, 2, 3)]. To successfully achieve the desired targets for the SDGs (4), we must also consider eight global existential challenges (GECs), or so called “wicked problems” [*sensu* (5)], which inextricably challenges humanities existence on this planet.

These eight GECs include: (1) Soil Security; (2) water security; (3) food security; (4) energy security; (5) climate change abatement; (6) biodiversity protection; (7) ecosystem services delivery; and (8) safeguarding human health (6). It is important to note that progress toward addressing these existential global threats is also critical to minimizing the rate of transgression of planetary boundaries (7, 8).

**Figure 1:**
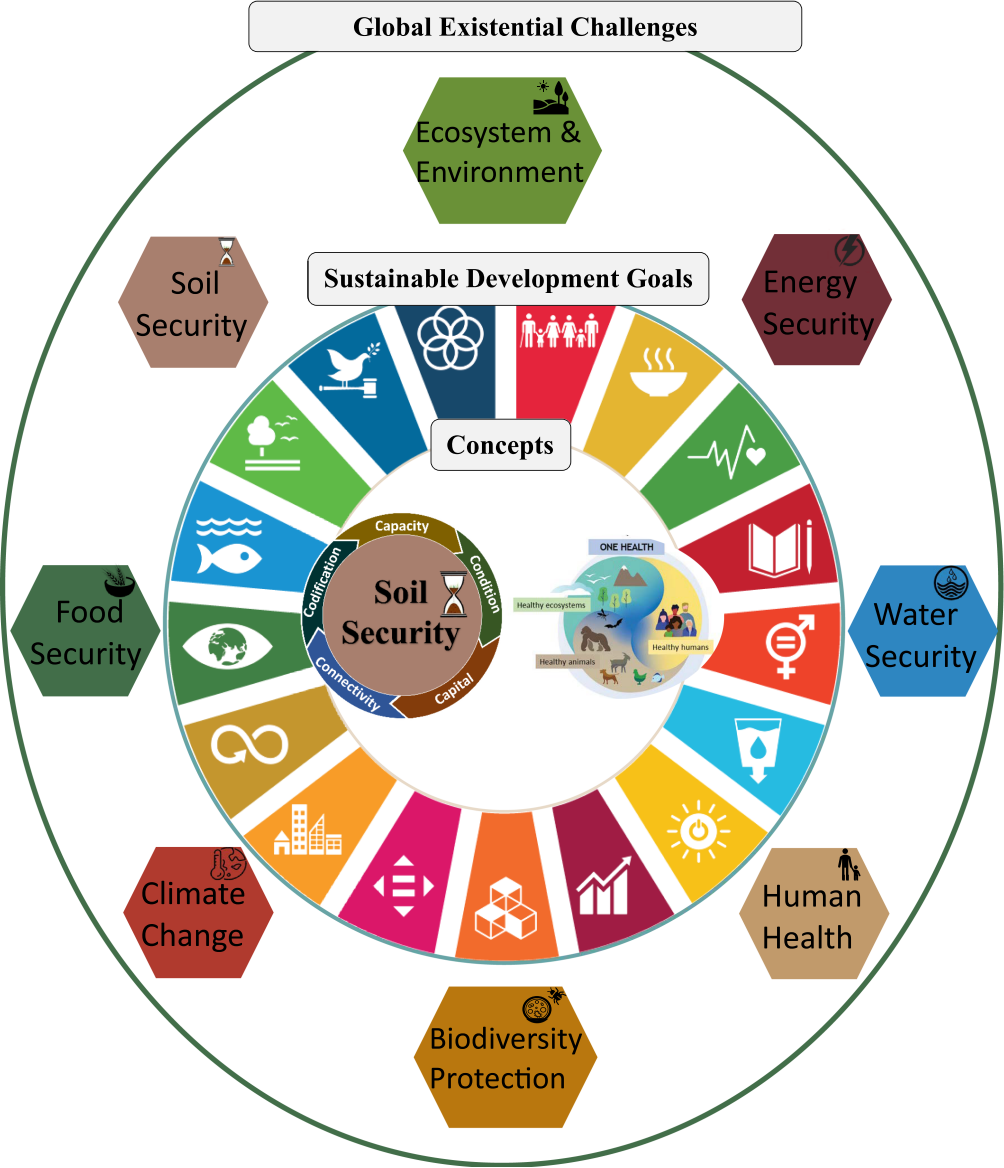
The five dimensions of Soil Security (capacity, condition, capital, connectivity, and codification) and the three components of One Health (human–animal–environment interface). Proposed linkages are explored between Soil Security and One Health (inner circle), as they relate to both the 2030 sustainable development goals (middle circle) and the eight global existential challenges (outer circle).

**Figure 2:**
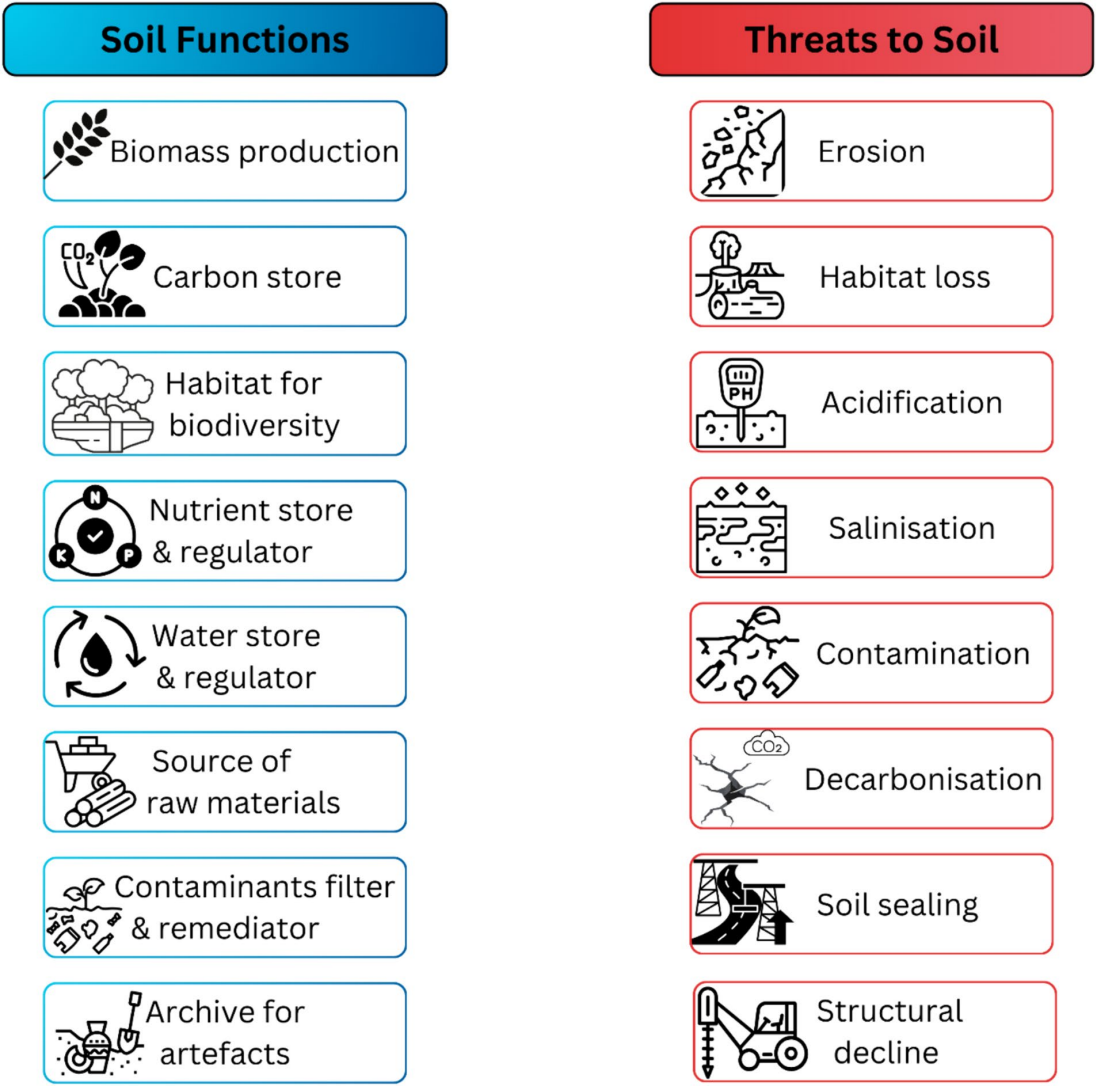
The eight key soil functions and threats to soil [as defined by Evangelista et al. (40)].

It is under this context that the concept of Soil Security emerged in 2012 (9–12). The vision of Soil Security is to act as a key contributor to help alleviate these global threats, through instilling the protection of important soil functions (e.g., the provision of food and the regulation of carbon) and for advocating policy (6) (Figures 1, 2).

Soil Security refers to “the maintenance and improvement of the world’s soil resource to produce food, fiber and fresh water, contribute to energy and climate sustainability and maintain the biodiversity and the overall protection of the ecosystem” (9). As the planet’s soil resources continue to be degraded (9, 13), this and other issues are increasingly publicized on mass media [e.g., “Kiss the Ground” documentary (14)] and other outlets. In turn, this publicity has and will likely continue to contribute to lobbying, policies and laws about soil. Presently, national and continental-scale public policies and legal frameworks concerning soil are gaining traction, with a particular emphasis on soil condition (i.e., Soil Health) in regions such as Australia and Europe (6).

The Soil Security concept is motivated by sustainable development and is driven by the need to: (1) secure food and fiber production that is not only productive, but profitable; (2) preserve biodiversity; and (3) contribute to water and climate sustainability. All these factors are critical to both the health of humans and the planet (15–17).

The Soil Security concept has potential linkages with several holistic and interdisciplinary concepts that work under the human–animal–environment interface. Three of the most popular concepts are: One Health, EcoHealth and Planetary Health (18). All three concepts differ slightly conceptually, theoretically and in their fields of usage (18–20). Out of all three concepts, the One Health concept and framework appears to offer the most global acceptance and engagement, with a quadripartite alliance between Organizations—the Food and Agriculture Organization of the United Nations (FAO), the WHO, the World Organization for Animal Health (WOAH) and the United Nations Environment Program (UNEP) (21, 22). Finally, of relevance to this perspective, is that a One Health approach (Figure 1; Box 1) has been called upon to help achieve the 2030 SDGs (23, 24).

BOX 1 **The new definition of One Health and a graphical representation of this definition. This definition has been adopted by the Quadripartite (WHO, WOAH, UNEP, and FAO) in 2022. Figure modified from Adisasmito et al. (58)**

Definition of One Health as developed by the One Health high-level expert panel:

“One Health is an integrated, unifying approach that aims to sustainably balance and optimize the health of people, animals, and ecosystems. It recognizes the health of humans, domestic and wild animals, plants, and the wider environment (including ecosystems) are closely linked and interdependent. The approach mobilizes multiple sectors, disciplines, and communities at varying levels of society to work together to foster wellbeing and tackle threats to health and ecosystems, while addressing the collective need for healthy food, water, energy, and air, taking action on climate change and contributing to sustainable development”

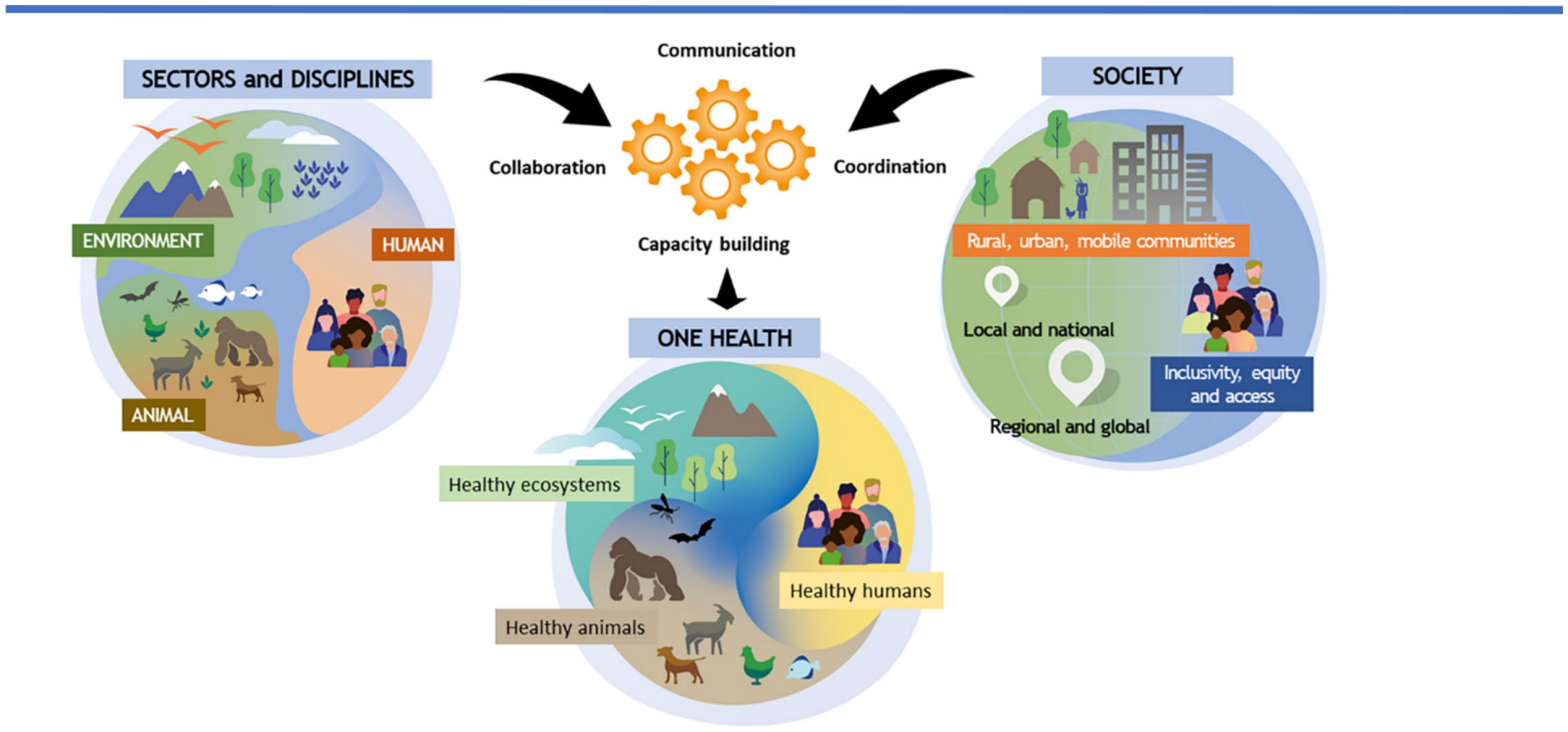

The relationship between Soil Security and One Health remains largely unexplored, despite the notable perceived overlap in objectives between these concepts. One example (see Box 2 for more examples) is that soils are home to more than 25% of global biodiversity (25) and more than 95% of the world’s food comes from soil (and soil organisms) (16); thus soils are vital for sustaining the health of humans, animals, ecosystems, and the planet—a key goal of One Health.

BOX 2 **Real-world examples linking Soil Security with One Health (human–animal–environment interface)**

**1 Nutrient cycling and food production**
  Soil provides essential nutrients for plant growth, which directly effects food quality and nutrition. Assessing soil nutrient levels and understanding nutrient cycling can help optimize agricultural practices and improve food security.
**2 Contaminants and health risks**
  Soil can contain harmful substances like heavy metals, pesticides, and pathogens.
  Studying soil contamination and assessing exposure pathways (e.g., ingestion and inhalation) may result in improved management of health risks.
**3 Soil microbiome and health**
  Soil hosts diverse microbial communities (e.g., bacteria, fungi and viruses) that influence human health. These microbes are essential for maintaining soil condition, which in turn affects animal and human health. Beneficial soil microbes assist in breaking down organic matter, cycling nutrients, and suppressing soil-borne diseases.
**4 Antimicrobial resistance**
  Antimicrobial resistance (AMR) is a global health issue that affects humans, animals, and the environment. Maintaining good soil condition is crucial in preventing the spread of AMR by maintaining a balanced microbial ecosystem. Protecting soil helps suppress harmful pathogens and supports food security and human health.
**5 Soil erosion and water quality**
  Soil erosion affects water quality by releasing sediments and pollutants. Assessing erosion rates and implementing soil protection measures (e.g., cover crops and terracing) is of immense benefit to soil, humans, animals and the environment.

The aim of this review is to investigate potential linkages between these two concepts and related themes. First, we outline the evolution and definitions of the two concepts. Second, we discuss where and how these concepts are used in the literature. Third, we assess the synergies and tensions of these concepts and outline our opinion on the nexus between Soil Security and One Health. Fourth, we proffer five measurable dimensions for One Health, to allow for an overall measure of One Health. Lastly, we synthesize One Health and Soil Security as they both relate to the 2030 SDGs and GECs.

## What both Soil Security and One Health represent

### Soil Security

Human knowledge of soil has historically been intrinsically linked with agriculture (26). In the first half of the 20th century, the erosion of agricultural soil prompted a growing awareness of the importance of soil conservation. Efforts were concentrated on devising strategies to mitigate soil loss caused by land use practices and to minimize the resulting negative effects on productivity and the economy (27–29). Much of this soil conservation impetus was derived from the devastating “Dust Bowl” phenomenon in the 1930s, occurring between Texas and Nebraska in the United States of America, resulting in extensive damage and harm to crops, cattle and people (30). This phenomenon was considered one of the most severe environmental crises in North America in the 20th century, resulting in estimated losses of over 480 tonnes of top soil and US $400 million in annual productivity (31). The principle causes of the “Dust Bowl” are attributed to dry farming techniques and severe droughts (32).

Since the 1970’s, land evaluation has provided information on the suitability of land for specific purposes (33). Over the following two decades, an increase in the quantity and speed of spatial data resulted in quantitative methods of land evaluation. However, these quantitative methods, largely driven by soil scientists, took little or no account of ecosystem, economic or social factors of land use (34). As the focus moved to addressing soil related issues beyond the limitations of agriculture, the soil care concept emerged, focused on sustainable land use requiring a simultaneous application of socio-economic concerns aligned with environmental management of soil (35). Since this time, a suite of soil-related concepts have emerged including: soil quality, Soil Health and soil protection [for a review of these concepts and others, see (6)]. The distinction between soil and land, along with a focus on soil functions and the recognition of eight threats to soil (Figure 2), lead to the proposal of soil protection (36). It is apparent that the Soil Security concept did not develop in isolation. Rather, when introduced in 2012, Soil Security encompassed other soil value and care concepts. Like soil protection, Soil Security adopts a soil-centric approach. Importantly, Soil Security relates to the need for soil functions to be protected on the same level as other human rights and provides a conceptual framework for addressing GECs (9). This viewpoint promotes that soils should be regarded as a common good (9)–similar to air and water–rather than treated only as private entities (37, 38).

**Figure 3:**
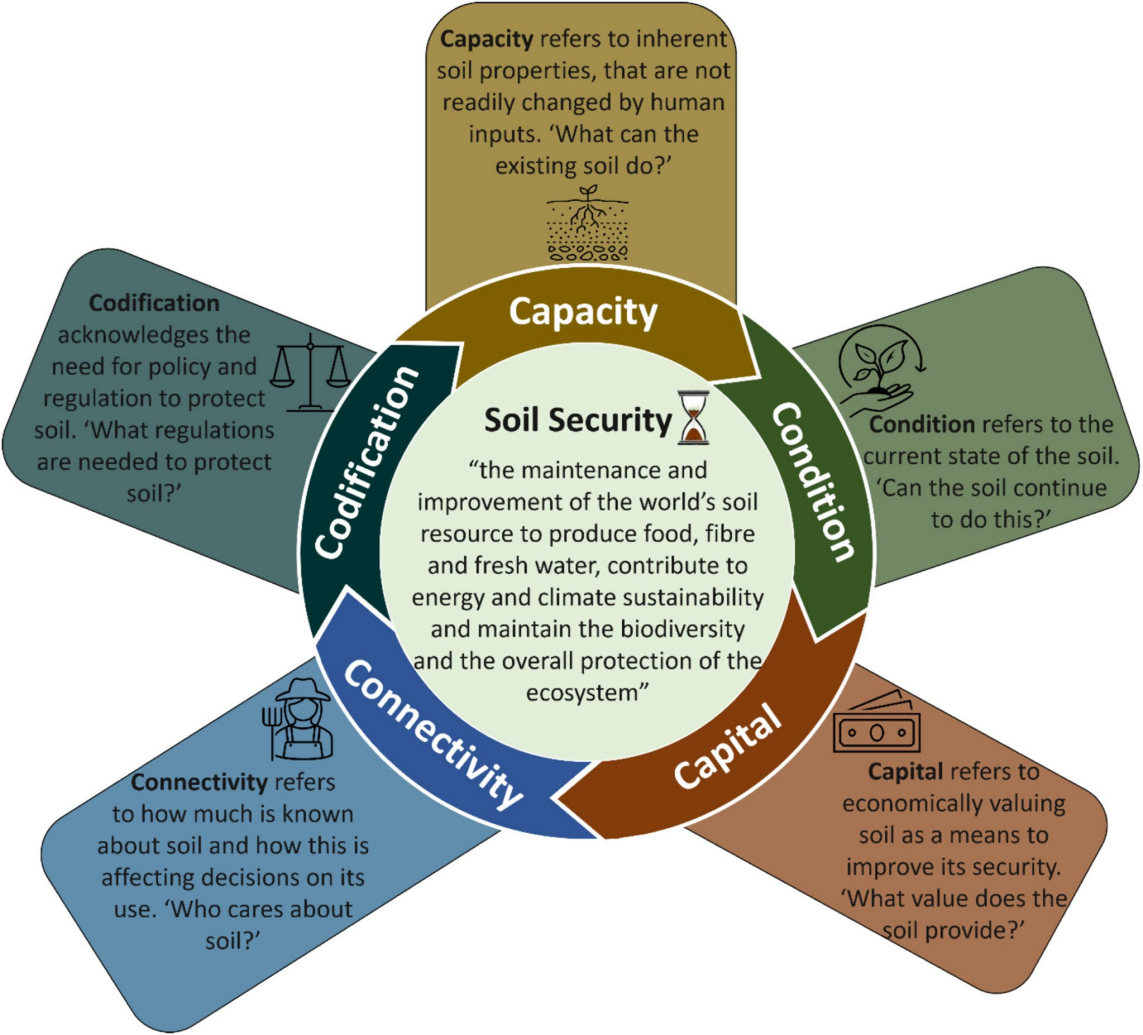
The five dimensions of Soil Security and the meaning for each dimension. Impediments in any one of these dimensions can challenge Soil Security.

Soil serves multiple functions, and an evaluation is essential to comprehend its capacity to fulfill these functions (39). There are five dimensions that frame Soil Security (Figure 3). Dimensions refer to the biophysical aspects of soil, such as the *Capacity* and *Condition*, in addition to the socioeconomic factors influencing those physical aspects such as *Capital, Connectivity,* and *Codification* (10). Each dimension is conceptualized to provide the means for assessment, comparison and to provide an accurate measure of Soil Security (15, 40).

Formal Soil Security assessment is gaining momentum (40). Early assessments have been undertaken in locations in Australia (41, 42), Papua New Guinea (43) and China (44). More recent publications have proposed a comprehensive list of indicators and analytical approaches that could be considered in developing more universally accepted future assessments protocols (6, 40). By being more informed about the current state of soil, we can plan for its optimal future use, and ensure the most beneficial management practices are followed for the soil and its plethora of users.

### One Health

The term “One Health” is relatively new, but the concept is ancient, dating back to BCE [as discussed elsewhere in (19, 45, 46)]. In brief, it has been argued that the ancient One Health concept has no singular origin in human thought (45). Rather, throughout human history this concept has been recognized as a fundamental condition for life on earth, repeatedly re-discovered and further explored by numerous historical figures (45). In BCE times, Hippocrates (460–367 BCE) and Aristotle (384–322 BCE) laid early foundations for One Health. Hippocrates highlighted the link between public health and a clean environment, while Aristotle introduced comparative medicine through his works on animal diseases (47). Over the next 2,500 years, figures like Giovanni Maria Lancisi (1654–1720), Claude Bourgelat (1712–1779), Louis-René Villermé (1782–1863) and Alexandre Parent-Duchatelet (1790–1835) furthered the understanding of the interdependence between animals, humans, and the environment.

In later centuries, Rudolf Virchow (1821–1902) coined the term “zoonosis,” inspiring physicians like William Osler (1849–1919) to integrate animal and human health through comparative biology and medicine (45). In the 20th century, James Steele and Calvin Schwabe pioneered the ecological approach to health (45). Schwabe popularized the term “One Medicine” in his book, Veterinary Medicine and Human Health, reflecting the ongoing integrative thinking in comparative medicine (48).

In the 21st century, One Health underwent rebranding with various agendas, alliances, and stakeholders involved [as discussed comprehensively in (45, 46, 50)]. In the 2000s, “One Medicine, One Health” focused on medical and veterinary collaboration. Concurrently, at the 2004 Wildlife Conservation Society conference, the term “One World, One Health” emerged, emphasizing an interdisciplinary approach to address interconnected threats to human, animals, and ecosystems. Toward the late 2000s, these agendas coalesced into a single banner of “One Health” (49). In this revitalized One Health approach, there is a strong emphasis on systems thinking, and collaboration across disciplines such as: veterinary and human medicine, public health, ecohealth, and more recently, planetary health (20, 50).

However, in the last decade especially, questions and critiques of One Health have emerged in response to the persistent anthropocentric focus (51, 52), colonial agendas and neo-liberal global health governance (53, 54) and lack of indigenous viewpoints (55)–to name but a few critiques. Likely because of the devastating COVID-19 pandemic (56), and partly in response to some, but not all of the above criticism [see (57) for a proposed paradigm shift of One Health], a new definition of One Health was declared in 2022 (Box 1) (58). This new definition aspires to shift the traditional One Health focus from zoonoses and antimicrobial resistance, to the full spectrum of health, including the health of the planet (58). If actualized, this new definition and direction could result in meaningful, collective action toward addressing both the SDGs and GECs. However, only time will tell whether this theoretic aim is realized in praxis.

## Methods and results

### Where are Soil Security and One Health concepts used and applied in the literature?

#### Literature search strategy

Guidelines and recommendations for the literature search and bibliometric analysis (specifically “keyword co-occurrence networks”) were provided by (59) and (60).

To better understand the evolution of Soil Security and One Health, we produced keyword co-occurrence networks in the large multidisciplinary database, Dimensions (61). The search was finalized on August 28, 2023, using the search terms “Soil Security” OR “One Health” OR “Onehealth.” The search option, “title,” which searched for these search terms in the title of a document was used. We used the “publication year” filter to include only records published between 2012 and 2023 and filtered these results to include only articles, chapters, or books. This date range was chosen to coincide with the first published usage (2012) of the Soil Security concept (12). These search results were explicitly designed to retrieve only relevant records of either Soil Security or One Health. Only records that were published in English were selected.

### Search limitations

This analysis was based solely on the Dimensions database. Although, previous studies have fortified the reliability and validity of both journal coverage and article retrieval between Dimensions and other databases (such as Scopus and Web of Science) (62, 63), it is possible that some relevant Soil Security and One Health records may have been excluded (e.g., articles which did not explicitly mention these search terms in the title of the publication and nonpeer reviewed material, such as reports). In addition, only the terms “Soil Security,” OR “One Health” OR “Onehealth” were used as search terms to generate the dataset used in this analysis and results may differ if using different search terms and databases. However, we deliberately chose to limit our search terms to reduce “false positives” (i.e., articles which did not explicitly utilize a Soil Security or One Health framework; of which there are scores of records) and using a single database provided us with a consistent dataset.

### Search results

Articles were screened for relevance prior to inclusion. The search on relevant One Health papers yielded 3,467, which were all included in the analysis. While the search on relevant Soil Security papers yielded 66 publications, which were all included in the analysis.

**Figure 4:**
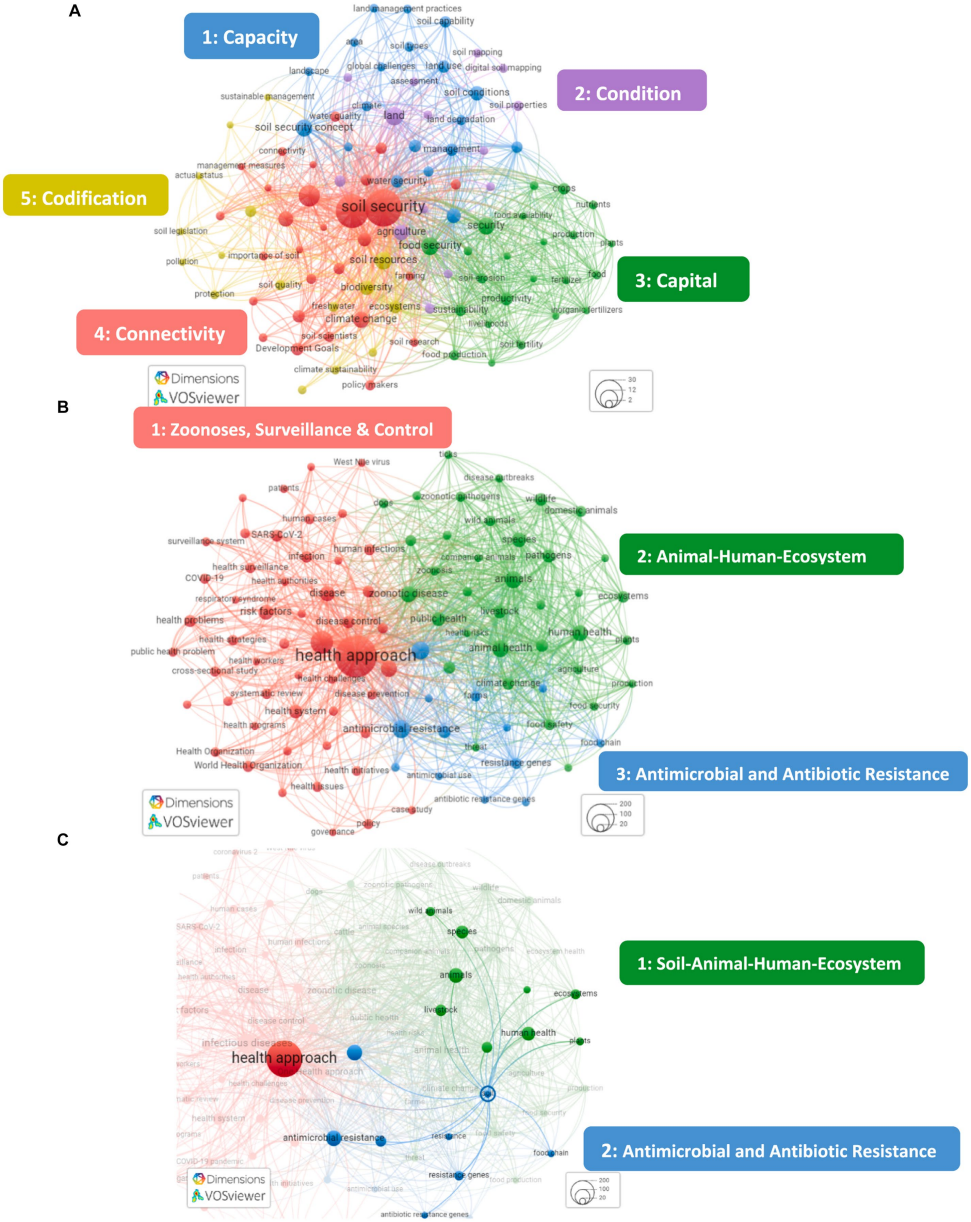
Keyword co-occurrence networks of Soil Security **(A)**, One Health **(B)** and possible linkages between Soil Security and One Health **(C)** research topics. The size of the nodes (scale: bottom right corner) represents the number of publications in which a keyword occurs (i.e., a larger node represents more common keywords). The closer the nodes to each other, the stronger the link between the keywords. The keywords are grouped in clusters depending on their inter-relation, and these clusters are represented with different colors. For each colored cluster, the following themes are labeled (inset). Graphs were created using VOSviewer online and dimensions database.

The keyword co-occurrence networks showed that the most represented topics in Soil Security publications related to agronomy, agriculture, and climate change, with keyword co-occurrences falling under one of the five Soil Security dimensions (Figure 4A). Conversely, for One Health publications, the most represented topics related to: (1) zoonoses, surveillance and control initiatives and measures; (2) the animal–human–ecosystem interface and; (3) antimicrobial and antibiotic resistance (Figure 4B). There were no publications which explored the linkage between “One Health” and “Soil Security.” However, there were publications which explored a link between the keywords “Health approach” and “soil” (Figure 4C). From these two keywords, two clusters emerged: one concerned with antimicrobial and antibiotic resistance in relation to soil, the other the soil–human–animal–ecosystem nexus (Figure 4C). The two clusters in Figure 4C will be reviewed, followed by a further exploration of the linkages between Soil Security and One Health.

## Soil Security—One Health linkages in the literature

### Antimicrobial resistance

Antimicrobial resistance (AMR) is a broad term which entails the microbial resistance to a wide range of antimicrobial agents, including antifungals, antibiotics and antiparasitic drugs (64). AMR is considered one of the most serious global public health threats of the 21st Century, as it threatens our ability to treat common infections (65). As a result of the widespread usage of antibiotics for both agriculture and human usage (66), AMR genes have spread rapidly in the environment, promoting soils as a sink for AMR. For example, experiments have shown that AMR genes can be transferred from contaminated manure in soils to vegetables (67), posing health threats to consumers. Conversely, soil is also a natural source of a plethora of AMR genes, the presence of which is utilized by microorganisms in competition and survival (68). In brief, although AMR has a negative effect on human health, the effects on Soil Health are unclear and require further investigation (69). Nonetheless, the protection of soil biodiversity and the microbes contained within, is an essential buffer in reducing the spread of AMR (70). In addition, soil biodiversity has intrinsic value as a source of new pharmaceutical compounds; as highlighted through the discovery of penicillin from a soil fungus and tetracycline from a soil bacteria (71). Finally, there is a growing body of literature linking soil and the human gut microbiome (72, 73). Soil is an inoculant and source of numerous beneficial human gut microorganisms; of high relevance to connecting healthy and diverse soils with One Health (69, 74).

In summary, this linkage is concerned with strategies to combat AMR, from both a health and soil perspective. Globally, One Health approaches have long been heralded by the WHO to combat AMR (75). However, fundamental knowledge about how the soil environment, soil type, soil properties, and crops are connected to AMR, is lacking (70). This presents a potential nexus that the concepts of Soil Security and One Health could work toward.

### Soil–human–animal–ecosystem nexus

The soil–human–animal–ecosystem nexus underscores the idea that actions affecting one component can have ripple effects on other components in the system. For example, poor soil management practices in agriculture can result in soil degradation, reduced yields and food insecurity; all of which can directly affect the soil, humans, animals and the ecosystem at large (39, 76). Numerous studies have investigated soil-related nexuses, under a variety of topics, between soil and related components (39, 77, 78). However, fewer studies have focused explicitly on soil under a One Health framework, which encompasses assorted nexuses as they relate to One Health. A related concept to Soil Security, Soil Stewardship, has previously been proposed as a nexus between ecosystem services and One Health (79). We argue that Soil Security is a broader term and encompasses Soil Stewardship, using the Soil Security dimensions of Capital and Condition. Soil Security argues for both soil functions and ecosystem services to be on the same level as human rights (9); central to advocating the protection of soils in policy.

In summary, the soil–human–animal–ecosystem nexus is concerned with assessing and improving the interrelated connections between components. It is worth noting that previous scholars have argued that food production should not only focus on producing quality, nutritious food, but also simultaneously restoring and sustaining the health of the soils (80, 81): the planet’s life support. This important argument typically come from the focus of solely Soil Health, which is encapsulated by the *Condition* dimension of Soil Security (40). By focusing on broader terms like Soil Security and One Health, it might not only be possible to improve Soil Health, but also sustainably balance and optimize the health of people, animals and ecosystems. The Soil Health concept presents a potential connector between the concepts of Soil Security and One Health.

### Other possible linkages between Soil Security and One Health

It is not surprising that “One Health” and “Soil Security” are absent in the keyword co-occurrence networks. To date, prominent research themes for “One Health,” have investigated human and animal health, specifically zoonoses, largely focusing on antimicrobial resistance and emerging infectious diseases (Figure 4B) (20, 82). Given that One Health was pioneered by medical and veterinary scientists (45), these prominent research themes are expected. The reported outcomes of One Health transdisciplinary research investigating the environment (or ecosystem) within the animal-human interface is seldomly investigated [but see (86)], and has repeatedly been called for further attention (83, 84). In addition, there has been growing recognition across different scientific disciplines that both soil chemistry, Soil Health (including soil microbes) and plant health form cornerstones of One Health approaches and should be explicitly included in One Health policy (69, 74, 85–89). We argue that Soil Security encompasses these concepts at the broadest scale and further to the above pleas, we advocate that Soil Security should also be explicitly included in One Health.

The next section will discuss the dimensions of both Soil Security and One Health.

## Discussion and theory formation

### Dimensions of both Soil Security and One Health

In addition to the new definition of One Health proposed in 2022, an over-arching Theory of Change (ToC) was also developed to provide: (1) an assessment on the emergence of health crises from the human–animal–ecosystem interface; and (2) to develop a long-term strategic approach to reducing the risk of zoonotic pandemics (90). The One Health ToC was designed to provide a conceptual framework linking numerous elements, including: (1) problem statements; (2) the approach and pathways of change; (3) high level actions; and (4) impacts, outcomes and the links between these steps. Central to the implementation of the ToC, are four so-called “dimensions” proposed to assist in the implementation of One Health approaches (58). The “4Cs”: communication, collaboration, coordination and capacity building, serve as the “cogs” between sectors and disciplines and society; moving from One Health theory to practice (Box 1). However, unlike the dimensions of Soil Security, the “4Cs” of One Health do not actually provide any measurements, such as the state of health in a given situation or sector (i.e., human, animal or environment). In this sense, the 4Cs are not measurable dimensions, rather they serve as actions, approaches or “operational dimensions” (91) to One Health facilitation. We believe that this is a major shortcoming of One Health. By omitting measurable dimensions, how can an assessment of whether One Health is functioning as an evidence-based approach, be undertaken?

In addition, there is little point talking about One Health, if we cannot or do not measure it, or understand how “healthy” each component (i.e., humans, animals and the environment) can be. Similarly, it is important to note that health cannot be solely measured as the absence of disease [*sensu* (92)], but rather that both disease and health are products of complex systems and interactions (93).

Recently, a potential overall index of One Health was proposed (94). One part of this index is the global One Health Intrinsic Drivers index (GOH-IDI) (95), which provides a single score for the One Health performance of countries. The GOH-IDI scored 146 countries on weighted indices across the human–animal–environment interface, producing overall One Health scores and rankings for each country (95). Although, a potentially useful index for international comparisons, there was no soil-related indicators (e.g., carbon indicators of Soil Health) used to determine this index. To truly align with the definition of One Health (Box 1), it is necessary to include soil-related indicators in this index. Finally, as previously mentioned, there is a clear need to define and conceptualize each One Health dimension, similar to how it has been accomplished for other concepts like Soil Security (40) and through the continuous evolution of dimensions in Food Security (96). Clearly defined dimensions can streamline the assessment, comparison, and measurement of One Health itself.

### Five measurable dimensions of One Health

There is a small body of literature discussing dimensions of One Health (97–99). In fact, an important point of difference between Soil Security and One Health, is concerning the definition of “dimensions.” For Soil Security, a dimension is a measurement (in the mathematical sense), to provide the means for assessment, comparison and to provide an accurate overall measure of Soil Security (15, 40). Examples include, biophysical aspects of soil, such as land capability assessment (Capacity) and a measure of soil organic carbon (Condition) (40). In addition, socioeconomic factors influencing the soil can also be measured, including: carbon credits (Capital), growers knowledge of soil (Connectivity) and the regulatory policies related to fertilizer and pesticide application and usage (Codification) (40).

In contrast to the definition of dimensions for Soil Security, One Health dimensions have been defined “as spaces in which levels of Organization occur” [*sensu* (98, 100)]. Examples of these include: geographic space, time, governance, economic, value, faith and linguistic dimensions (97, 98). In addition, the scale (e.g., local–national–global) or level (e.g., microbial–individual–population) of these dimensions are considered (97, 98). While acknowledging the inherent complexity and the above described multi-faceted aspects of One Health (97, 98), we consider that these One Health dimensions need to also contain an explicit measurable component, like dimensions of Food Security (96) and Soil Security (40). With this in mind, we proffer five measurable dimensions for One Health, the “5Cs”: Capacity, Condition, Capital, Connectivity, and Codification. These dimensions have been adopted from Soil Security and aim to encompass both the biophysical and socioeconomic facets of One Health.

It is important to note that our view of health, aligns with that of the WHO, defining health as “a state of complete physical, mental, and social wellbeing, not merely the absence of disease or infirmity” (92). Under this definition, we provide possible indicators for each proffered dimension of One Health. These indicators are illustrative only and by no means exhaustive. Below, we briefly define each dimension as it relates to the three components of One Health: human–animal–environment interface.

## Dimensions of One Health: the “5Cs”

### Capacity

The Capacity dimension, refers to the inherent health properties of each component: “what is the existing health of the component or species?” Examples include: (1) human—disability adjusted life years (DALY) and the GOH-IDI; (2) animal—biodiversity indicators (e.g., Shannon’s diversity); and (3) environment—air quality measurements.

### Condition

The Condition dimension refers to the current health of each component or species, relative to a comparable reference component, species or population (at a comparable age and stage): “can this component or species continue to fulfill this function?” Examples include: (1) human—body mass index or blood pressure measurements; (2) animal—effective population size (N_e_) and diversity indices (i.e., Beta and Gamma diversity); and (3) environment—relative ecosystem health measures, including indicator species (as evidence for environmental change) and diversity indices.

### Capital

The Capital dimension, refers to economically valuing health as a means to invest: “what value does the health of this component, species, population or service provide.” A monetary unit metric, such as a cost–benefit analysis (CBA) or cost-effectiveness analysis (CEA) are both highly appropriate (101). A CBA assigns costs to all monetary and non-monetary (e.g., wellbeing) outcomes for evaluation (e.g., CBA for initiating a mining lease). In contrast, a CEA identifies the most cost-effective option, expressed in terms of monetary cost per unit (e.g., fertilizer cost to increase crop yield). As CBA requires the assignment of costs to all outcomes, a CEA is likely more appropriate in circumstances where components have factors that are difficult to monetize (e.g., environmental outcomes from CO_2_ mitigation). Possible examples for One Health components include: (1) human—CBA and CEA for individual patient care and healthcare systems; (2) animal—CBA associated with the loss of ecosystem services (e.g., insect pollinators); and (3) environment—as similar for (2) and also CEA for CO_2_ mitigation measures.

### Connectivity

The Connectivity dimension refers to measures of awareness or literacy: “what degree of human awareness is known about the components of health?” This is broadly considered health literacy, defined as “the ability of an individual to obtain and translate knowledge and information in order to maintain and improve health in a way that is appropriate to the individual and system contexts” (102). We extend this idea of health literacy to also include animal health literacy and environmental health literacy. Examples include: (1) human—scores of health literacy such as the European Health Literacy Survey (103, 104); (2) animal—knowledge of and awareness of animal stewardship, welfare, food chain and habitat provision (indicator example: World Animal Health Information System); and (3) environment—knowledge of and awareness of environmental, ecosystem and planetary concerns and stewardship, including climate change mitigation [indicator example: the New Ecological Paradigm Scale (105)].

### Codification

The Codification dimension refers to the degree of governance and regulation of health systems for each component, species or population. Examples include: (1) human—laws and policies influencing health care, health promotion and disease prevention (e.g., Tobacco and alcohol taxes and legislation); (2) animal—multilateral treaties for the protection of wildlife, such as the Convention on International Trade in Endangered Species of Wild Fauna and Flora (CITES); and (3) environmental laws and policies in place to protect, maintain and preserve the environment and associated ecosystem services (e.g., Environmental Protection Act).

By way of summary, a tabulated list of potential indicators by each dimension is provided in Table 1.

**Table 1:** The five proposed One Health dimensions (the “5Cs”) as they relate to the three One Health components (human–animal–environment interface) and examples of possible indicators.

	Dimensions				
	Capacity (the capacity of existing health)	Condition (the capacity of existing health, relative to a reference)	Capital (financial measures of investment in health)	Connectivity (health literacy; human awareness of health)	Codification (governance, laws and policies)
**One Health components**					
Human health	DALY, GOH-IDI*	BMI, blood pressure measurements and GOH-IDI*	CBA and CEA associated with expenditure costs in health attributed to individual and healthcare systems (Governments total spendings)	HLS-EU-Q and other health literacy indicators (104)	Laws and policies influencing health prevention, care and promotion (e.g., Tobacco, alcohol policies/laws)
Animal and ecosystem health and diversity	Biodiversity indices (e.g., Shannon’s diversity index)*, GOH-IDI*	Diversity indices (i.e., Beta and Gamma diversity), effective population size (Ne)* and GOH-IDI*	CBA associated with the loss of ecosystem services (e.g., insect pollinators)*	OECD health indicators* and WAHIS database	CITES* and GOHI-IDI.
Environmental and ecosystem health and diversity	Biodiversity indices, state of global air quality, GOH-IDI*	Relative ecosystem health (e.g., indicator species presence), including diversity indices (i.e., Beta and Gamma diversity)* and GOH-IDI*	CBA associated with the loss of ecosystem services*. CEA associated with environmental outcomes from CO_2_ mitigation measures.	NEP and climate change dashboard	CITES* and climate change dashboard (Government policy indicators)

The list of indicators is illustrative and not exhaustive. *Indicators which likely operate across multiple One Health components (e.g., diversity measures for animal and environmental health). DALY, Disability Adjusted Life Years; GOH-IDI, Global One Health Intrinsic Drivers index; BMI, Body mass index; CBA, cost–benefit analysis; CEA, cost-effectiveness analysis; HLS-EU-Q, European Health Literacy Survey; OECD, Organization for Economic Co-operation and Development; WAHIS, World Animal Health Information System; NEP, New Ecological Paradigm Scale; CITES, Convention on International Trade in Endangered Species of Wild Fauna and Flora.

Currently, there are no well-established and verified indicators to evaluate performance, value-added, trade-offs, or the positive and negative consequences of a One Health approach (106–108). We believe that the inclusion of measurable dimensions for the human–animal–environment interface of One Health is an imperative first step in this direction; to allow for the assessment, comparison, and measurement of multiple dimensions of One Health. As a proposed evolution of One Health seeks to engage multiple worldviews (57), we encourage and invite anyone who finds these dimensions useful to discuss and contribute to the development and refinement of these One Health dimensions.

The next section will discuss the synergies and tensions between Soil Security and One Health and the possible connector of Soil Health.

### The synergies and tensions between Soil Security and One Health

It could be argued that both Soil Security and One Health concepts are both attempting to utilize a transdisciplinary research (TDR) approach. A TDR approach recognizes the multi-faceted nature of real-world problems, which require more than one perspective to solve (109). A TDR approach can be defined as a mode of research which “involves actors from different societal domains to co-produce action-oriented knowledge, which has the potential to contribute to transformative change” (110). This is exemplified by the fact that Soil Security and One Health call for the participation of different actors and disciplines to provide a well-rounded understanding of the problem at hand (Figure 3). Research findings from both Soil Security and One Health are expected to provide impetus for members of the public and policymakers to act (6, 9, 22, 111). Table 2 provides examples of various Organizations using either aspects of Soil Security or One Health concepts and frameworks. The widespread adoption of both the Soil Security and One Health concepts by various national and international bodies is indeed a positive development. However, it is evident that these two concepts often operate independently in separate domains, indicating a degree of compartmentalization or “siloing.” In practice, it appears that the two concepts are treated as separate entities with limited integration. The example of the Food and Agriculture Organization (FAO), which appears to have teams that operate independently in both concepts (Table 2), illustrates just one potential challenge for successful integration between these concepts.

**Table 2:** Examples of projects and programs utilizing either aspects of Soil Security or One Health concepts and frameworks.

Concept	Examples	Usage	References
**Soil Security**	Food and Agriculture Organization (FAO): Global Soil Partnership (GSP).	Initiatives focus on: (1) advocacy for policies and raising public awareness at various levels; (2) the creation of technical resources for countries to produce their own soil data; and (3) the implementation of initiatives aimed at adopting sustainable soil management practices at the grower level.	(127) https://fao.org/global-soil-partnership/en/
	The U.S. Department of Agriculture (USDA)	Promotes Soil Health practices among farmers and provides resources to improve soil management.	https://www.nrcs.usda.gov/conservation-basics/natural-resource-concerns/soil/soil-science
	Australian Government, Department of Agriculture, Water and the Environment	Supports initiatives and research aimed at improving soil management and sustainability. This includes a National Soil Strategy (policy document) released in 2021, which aims to outline how Australia will manage and improve its soil over the next 20 years.	https://www.agriculture.gov.au/agriculture-land/farm-food-drought/natural-resources/soils
	European Union, European Soil Data Centre (ESDAC)	Adopted various policies and programs to address Soil Security and sustainability. The ESDAC is one of the initiatives that provide data and information to support soil management and conservation efforts.	https://esdac.jrc.ec.europa.eu/
	Korean Soil Information System (KOSIS)	Contributes to sustainable land use, efficient agriculture, and environmental protection in South Korea by providing accurate and up-to-date soil information to a wide range of users. This system’s integration of soil data and mapping has made it an essential tool for ensuring responsible land and resource management in the country, by supporting farmers to make effective decisions.	(128)
	African Soil Information Service (AfSIS)	Recognizes the importance of Soil Security addressing Food Security and environmental challenges on the continent. Specific objectives are to improve soil mapping and information for sustainable agricultural practices.	http://africasoils.net/
**One Health**	FAO, OIE, and WHO: global early warning system for animal diseases (GLEWS+)	A multi-sectoral framework for conducting collaborative risk assessments for devising risk management strategies for health events occurring at the interface of human–animal–ecosystems. Recent examples include, an evaluation of the risks associated with the introduction and transmission of SARS-CoV-2 within mink fur farms and the associated potential spillover from these farms to humans and vulnerable wildlife.	www.glews.net
	World Health Organization (WHO)	The development of global health policies and initiatives. Recent examples include policies related to Food Security the ongoing development of a pandemic treaty (129).	https://www.who.int/europe/initiatives/one-health (130)
	World Organization for Animal Health (OIE):	Promotes the One Health concept and collaborates with WHO and FAO to address zoonotic diseases and AMR.	https://www.woah.org/en/what-we-do/global-initiatives/one-health/
	National One Health Initiatives	Many countries (e.g., Australia, Canada, Thailand and Kenya) have launched national One Health initiatives that involve cooperation between government agencies, research institutions, and healthcare providers to address zoonotic diseases and other health challenges.	Australia example: Australian Centre for International Agricultural Research: https://www.aciar.gov.au/one-health
	Academic Programs: Universities and academic institutions	Numerous One Health programs and courses exist to educate the next generation of health professionals and researchers.	https://www.onehealthcommission.org/en/resources__services/whos_who_in_one_health/academic_organizations/
	Non-Governmental Organizations (NGOs)	Various NGOs work on One Health issues, including wildlife conservation, disease prevention, and public health.	https://www.onehealthcommission.org/en/resources__services/whos_who_in_one_health/nonprofit_or_coalition_organizations/

### Soil Health as the connector between Soil Security and One Health

**Figure 5:**
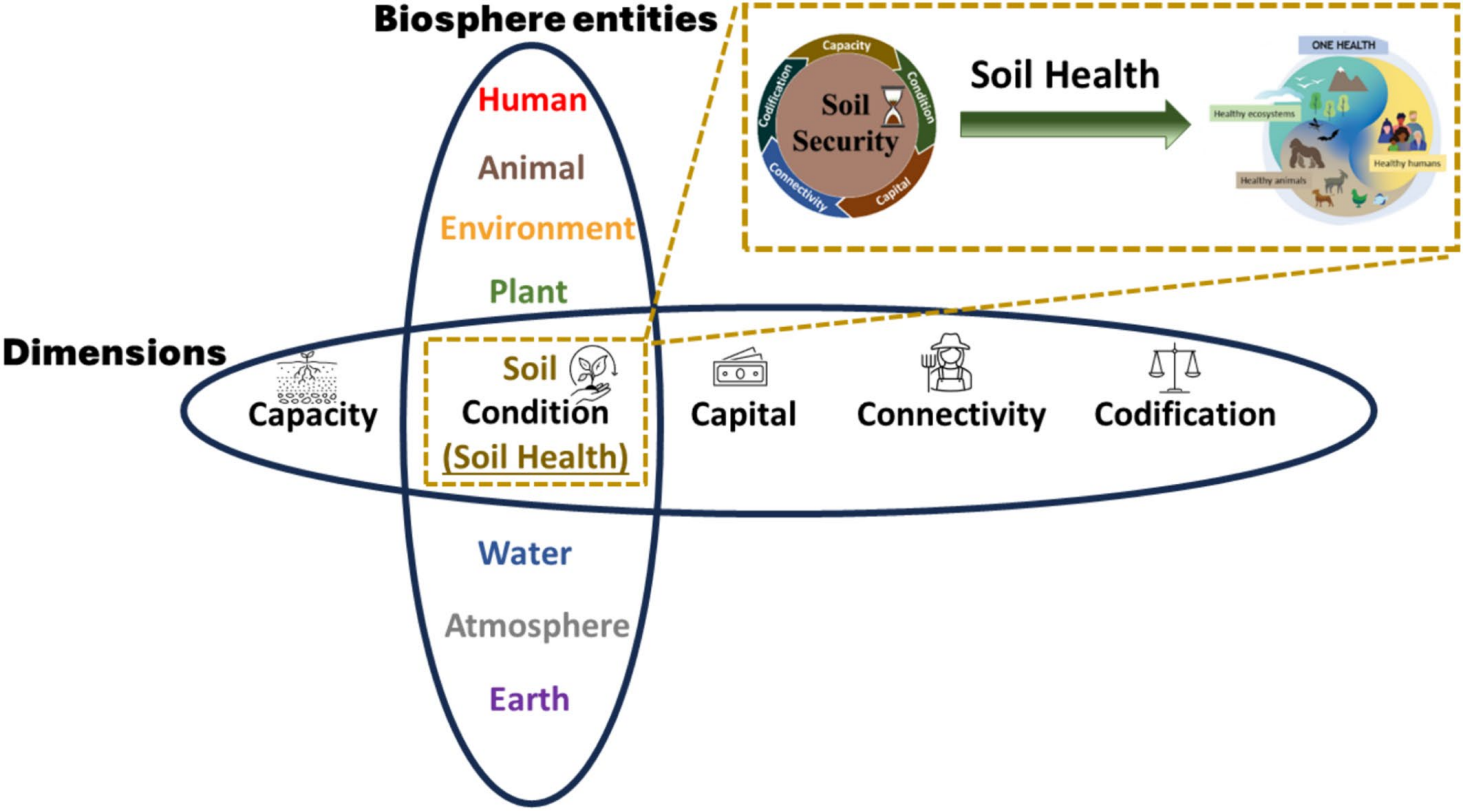
The proposed nexus between Soil Security and One Health. The overlap is focused on the concept of Soil Health (under the condition dimension in Soil Security), which connects Soil Security and One Health. Biosphere entities under One Health are selected examples. Top-right box represents a simplified version, depicting Soil Health as the nexus between Soil Security and One Health. See Figure 3 for definitions of each dimension.

Considering the challenge of integrating across disciplinary boundaries, we propose that the existing concept of Soil Health (the soil condition dimension in Soil Security) could serve as a useful connector between Soil Security and One Health (Figure 5).

Soil Health has previously been recognized under the Soil Security condition dimension (6). The Soil Health concept has widely been used in the field of soil science for around two decades, with the concept continually developing and evolving (38, 112). Importantly, Soil Health extends its focus beyond just the health of the soils, but also to the health of the planet (16, 38)–making it highly applicable to One Health.

Soil Health has been defined as the “ability of the soil to produce biomass, regulate the carbon pool, provide habitat for biodiversity, cycle nutrients, and cycle water” (16). The benefits ensuring and sustaining Soil Health are numerous. Healthy soils are fundamental for food safety, agricultural sustainability, and ecosystem health (16, 81, 86, 113), all of which are pivotal components of the One Health framework. In addition, the ability of soil to influence both disease transmission (114), AMR (70) and Food Security (39, 76) underscores its significance within the broader One Health context. Finally, the role of soils in sequestering carbon and mitigating climate change (115), aligns with One Health’s recognition of the complex environmental factors affecting human and animal health. It is under this context that we consider Soil Health a useful connector between Soil Security and One Health: by considering Soil Health as an integral component in the comprehensive pursuit of One Health goals (Figure 5).

In the final section, we will discuss how Soil Security and One Health concepts both relate to the 2030 SDGs and the GECs.

### Soil Security and One Health integration for the 2030 SDGs and GECs

To address the most urgent “global challenges” (116), such as climate change, poverty, inequality, and access to quality education, the UN adopted 17 ambitious 2030 SDGs (116). These goals, adopted by all 193 UN member states in September 2015, are intended to serve as a catalyst for action in vital areas that encompass equity, health and wellbeing for people and the planet. However, as of 2024, with just 6 years remaining until the 2030 deadline, it is disheartening to note that only ~15% of the SDGs are progressing as planned toward successful completion (117). It is possible that, the concepts of Soil Security and One Health could be useful in altering this trajectory.

However, currently, neither Soil Security or One Health are explicitly referred to in the 2030 SDGs (118, 119). There is a small body of literature discussing specific aspects of either soil-related approaches (1, 2, 3, 120), including Soil Security (118, 121), or One Health approaches (23, 24) toward gaining progress on the SDGs. However, as of 2024, it appears that these ideas and approaches are seldomly ever considered or discussed outside of their respective disciplines. This is possibly a result of the sectorial approach which SDGs are commonly placed, broadly divided into environmental (SDGs 6, 13, 14, and 15), social/political (SDGs 1, 2, 3, 4, 5, 6, 11, 16, and 17) and economic (SDGs 8, 9, 10, and 12) goals.

**Figure 6:**
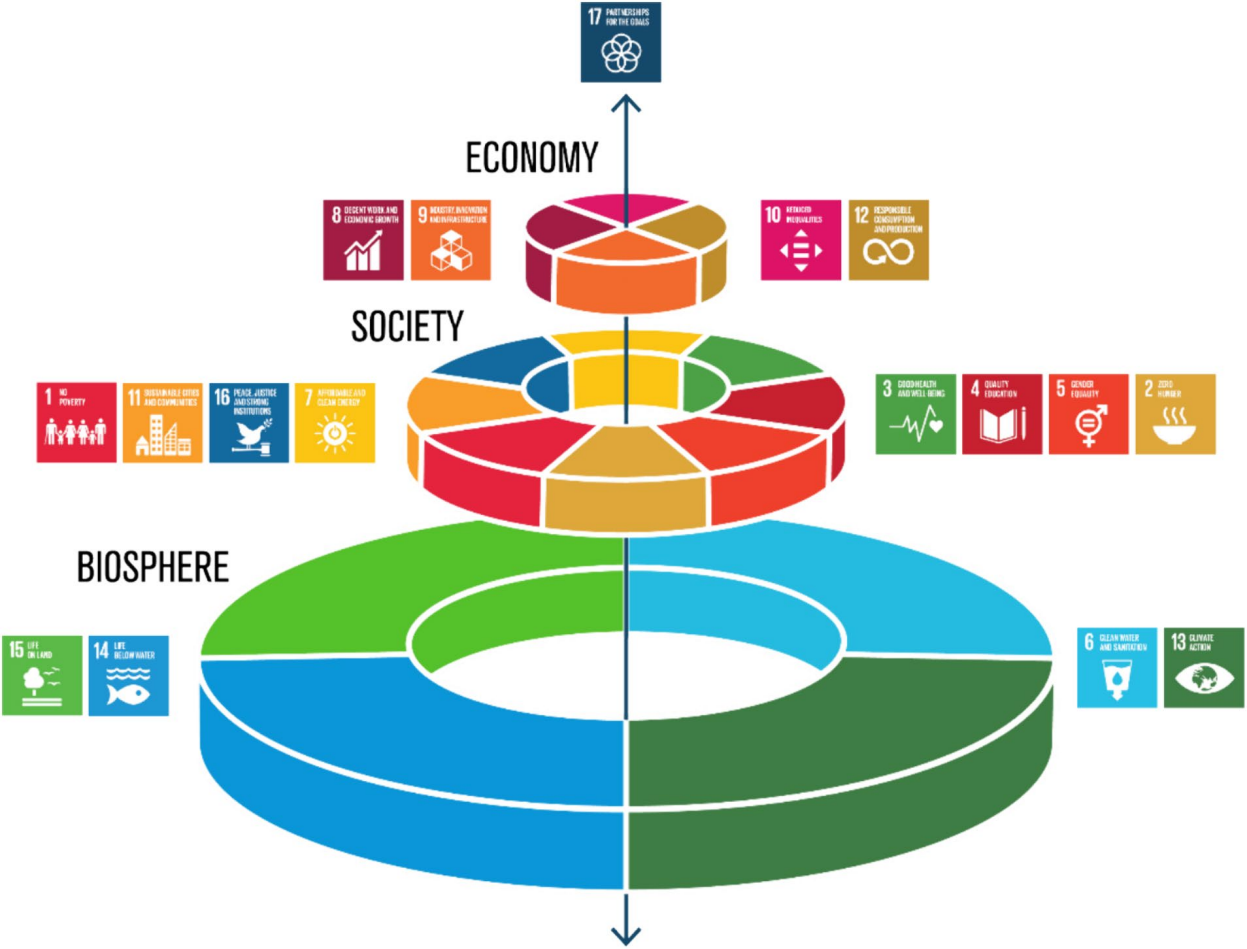
The Sustainable Development Goals (SDGs) wedding cake. The illustration of the SDGs wedding cake depicts the perspective that economies and societies should be considered integral components within the biosphere. This departs from the prevailing sectoral approach, where SDGs are broadly divided into environmental, social/political and economic sectors. Credit: Stockholm Resilience Centre, Stockholm University (CC BY-ND 3.0).

Perhaps, a more engaging approach of integrating the above viewpoints and the SDGs is through the hierarchical clustering of SDGs into three layers: the biosphere; society; and the economy. The SDG wedding cake framework is such an approach (Figure 6). Figure 6 depicts that economies and societies should be seen as embedded and dependent parts of the biosphere (122), defined here as the “global ecological system integrating all living beings and their relationships in the thin layer of life between the Earth’s crust and outer space” (123). Originally, this wedding cake framework was introduced to argue that SDGs are “directly or indirectly connected to sustainable and healthy food” (122). However, in a broader sense, this framework supports the assertion that the SDGs and life on the planet itself is embedded and dependent on the biosphere. This framework places much needed emphasis on maintaining the integrity of the earth’s life-support systems, or planetary integrity (124), of importance for maintaining a “safe operating space for humanity” (7, 8) and of relevance toward addressing the GECs (6). Indeed, through the application of the planetary boundaries framework (125), soil emerges as a pivotal factor in regulating essential Earth-system processes (17).

It is under this biosphere-focused SDG framework (Figure 6) that soil and its related functions are of critical importance. The contributions that soil makes to the biosphere is through its “functions” (Figure 2), defined here as “the ability of a soil to produce (and continue to produce) a particular outcome” (40). Crucially, soil is “multifunctional” as it performs a variety of functions contemporaneously, of critical importance to underpinning the health of humans and the planet (16). Previous studies have mapped soil functions to either ecosystem services (1) or ‘Nature’s Contributions to People’ (3, 120), with the delivery of these services or contributions then mapped on to delivering the SDGs. Our perspective supports these ideas and provides further impetus that the SDGs and life on the planet itself is embedded and dependent on the biosphere. It is under this context, that soil and its associated functions, fundamentally require Soil Security.

Finally, for One Health, with the UNEP joining the One Health Quadripartite in 2022 (58), there exists an opportunity for One Health to devote attention to the protection, maintenance and improvement of the biosphere and its many life-sustaining functions. For example, One Health governance could develop policies for environmental and health assessment (including Soil Health) into residential, industry and government development projects (126). In addition, educational programs aimed at increasing the agricultural literacy of health professionals, guided by Soil Security principles, could be implemented. We believe that by embedding a biosphere-focused framework into the *modus operandi* of the public, policymakers, and political leaders, will we then only see substantial progress on the 2030 SDGs and GECs.

## Conclusion

Overall, we argue that both Soil Security and One Health are highly complementary fields of scientific inquiry with solid leverage for translation into policy and practice. Our results provide the first visual representation of the Soil Security and One Health research fields and the interconnections between these fields. These results show that no integrative research between these two concepts has occurred. We suggest that the Soil Health concept is a useful connector between these two concepts. It is through the Soil Health linkage that we consider meaningful opportunities exist to build a stronger research community and extend transdisciplinary research approaches between these disciplines. Operationalizing and integrating Soil Security with One Health, through Soil Health, could greatly enhance ecosystem service delivery, public health, and agricultural sustainability (to name but a few examples). In turn, this may collectively result in progress toward the 2030 SDGs and the GECs. Finally, we have proffered five measurable dimensions (the “5Cs”), to allow for the overall measure of One Health. We encourage and invite anyone who finds these dimensions useful to contribute and critique to the further development and refinement of these One Health dimensions.
